# Myopericarditis in an emergency department patient presenting with chest pain and ECG changes: a case report

**DOI:** 10.1186/s43044-026-00734-7

**Published:** 2026-04-07

**Authors:** Lauren Coaxum, Colleen McClean, Rebecca Theophanous

**Affiliations:** Duke University Health System, Durham, USA

**Keywords:** Myopericarditis, Pericarditis, Myocarditis, Point-of-care ultrasound, Case report

## Abstract

**Background:**

Myopericarditis is uncommon but important to consider in patients presenting with chest pain. It most commonly is caused by a viral infection. Myopericarditis involves both myocardial injury and pericardial sac inflammation, thus may benefit from close monitoring and expedited treatment. It can present like pericarditis with chest pain, cold-type symptoms, diffuse ST elevations and PR depressions on electrocardiogram (ECG), and pericardial effusion on ultrasound. Risk stratification depends on case severity. More severe cases of myopericarditis can demonstrate elevated troponins and myocardial involvement, including potential left ventricular dysfunction.

**Case presentation:**

We describe an 81-year-old male patient with three days of pleuritic chest pain, dyspnea on exertion, and upper respiratory infection symptoms. He was evaluated by cardiology due to diffuse ST elevations on his ECG but did not have reciprocal changes, making acute coronary syndrome (ACS) less likely. His workup showed elevated inflammatory markers and elevated high-sensitivity troponin levels, indicating myocardial injury and distinguishing his diagnosis from pericarditis. His respiratory viral panel was negative. Bedside ultrasound showed a small pericardial effusion and moderately reduced ejection fraction (EF 40%). He was initiated on treatment for myopericarditis with colchicine and high-dose ASA (650 mg three times daily) for 3–4 weeks, with significant symptom improvement within 1–2 days.

**Conclusions:**

Emergency physicians should initiate early treatment for myopericarditis to reduce inflammation for improved patient outcomes while still considering ACS in all patients presenting with chest pain. Point-of-care ultrasound can identify a pericardial effusion or acute heart failure to guide management. Patients with myocardial involvement can develop permanent heart damage but can exhibit excellent recovery with appropriate treatment, which includes nonsteroidal anti-inflammatories, colchicine, and high-dose ASA.

**Supplementary Information:**

The online version contains supplementary material available at 10.1186/s43044-026-00734-7.

## Background

Myopericarditis is an uncommon diagnosis with myocardial injury plus pericardial sac inflammation [1]. Acute pericarditis comprises roughly 5% of emergency department (ED) visits for chest pain in North America and Western Europe and 0.2% of all cardiovascular hospital admissions annually [1, 2]. Myocarditis is more serious, with a mortality rate of 1–7% and causes elevated cardiac biomarkers or newly depressed left ventricular function on echocardiogram [1–3]. Myopericarditis is most commonly caused by viruses, including coxsackievirus, adenovirus, influenza, Epstein Barr, hepatitis C, and coronavirus disease (COVID-19) [1, 2]. Bacterial infections, vaccinations, pharmaceuticals, metastatic cancer, inflammatory bowel disease, and radiation are additional causes [1–2].

Per the 2025 European Society of Cardiology (ESC) and American Heart Association (AHA) guidelines, pericarditis diagnosis requires two or more of the following: pleuritic chest pain, pericardial friction rub on exam, electrocardiogram (ECG) changes, or new or worsening pericardial effusion (e.g. on echocardiogram, computed tomography (CT), or cardiac magnetic resonance imaging (MRI)) [1, 4, 5]. The most common presenting symptom is chest pain (82–95% of patients) [1–2]. Chest pain is typically pleuritic, worse when lying down, improved with sitting up or forward, and may radiate to the trapezius ridge [1, 2]. Patients may also have dyspnea (19–49%), coughing, hiccups, syncope (5–7%), or signs of systemic illness such as weight loss, night sweats, rash, or arthralgias [1–2]. The most specific ECG changes for pericarditis are diffuse ST elevations with PR depressions (stage 1). An additional early stage 1 finding in about 30% of patients with pericarditis (and 5% of ST elevation myocardial infarction patients) is Spodick’s sign, or a downsloping TP segment, best visualized in leads II and the lateral precordial leads [6]. Up to 60% of patients may present with transitional findings during disease progression including: J points on the baseline before T waves begin to flatten (stage 2), T wave inversion (stage 3), and normalization of the ECG (stage 4).^1^ 40% of patients with pericarditis can progress to myocarditis, which involves deeper myocardial inflammation [1]. ESC and AHS guidelines recommend early cardiac imaging with echocardiography to diagnose new or worsening pericardial effusion and rule out dangerous complications such as cardiac tamponade or constrictive pericarditis. Ultrasound can also diagnose ventricular dysfunction in cases of myocarditis [1, 2, 4, 5].

We describe a case of myopericarditis in an elderly ED patient using the CARE guidelines (for Case Reports).

## Case presentation

An 81-year-old male patient with paroxysmal atrial fibrillation and prior ablation two years ago (on rivaroxaban and dronedarone), chronic heart failure (ejection fraction or EF 45%), moderate mitral and tricuspid regurgitation, and ocular myasthenia gravis (under surveillance) presented to the ED with three days of pleuritic chest pain, sore throat, cough, rhinorrhea, and dyspnea. His pain worsened when lying down. He denied fever, leg swelling, vomiting, diarrhea, or abdominal pain. He had started doxycycline for bronchitis from urgent care the day before. He was tachycardic at 113 beats per minute, blood pressure 110/83mmHg, afebrile, respiratory rate of 20 breaths per minute, and 98% oxygen saturation on room air. His exam revealed a friction rub, tachycardia, regular heart rhythm, clear lungs with mild tachypnea, no abdominal tenderness, and no leg swelling or jugular venous distension (JVD).

**Fig. 1:**
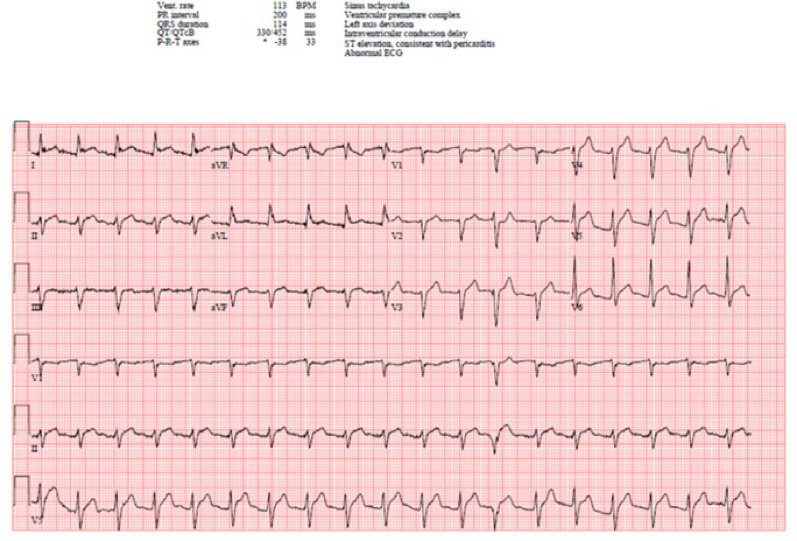
Electrocardiogram (ECG) with sinus tachycardia, diffuse ST elevations, but no reciprocal ST depressions or T wave inversions

**Fig. 2:**
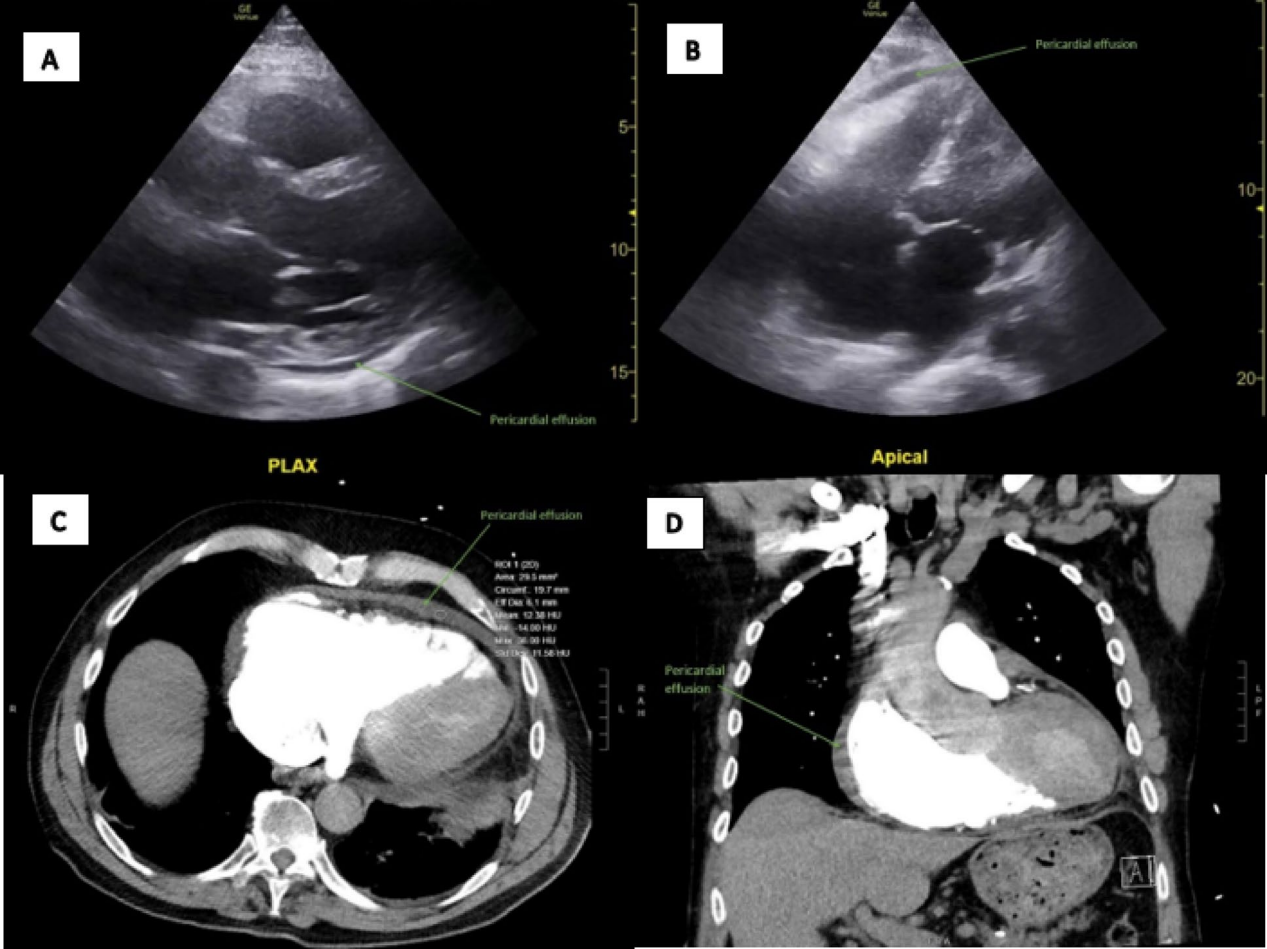
Panels **A** and **B**. Point-of-care cardiac ultrasound still image, parasternal long axis and apical four chamber views, showing a small to moderate pericardial effusion and dilated cardiomyopathy (B-mode). Panels **C** and **D**. Axial and coronal chest computed tomography scan with intravenous contrast showing a small to moderate pericardial effusion with mild pericardial fat stranding

ECG showed sinus tachycardia at 113 bpm, first degree AV block, and diffuse ST elevations without reciprocal changes (different from prior ECG) (Fig. 1). His blood work showed leukocytosis with 14.9 × 10^9 white blood cells/mL, hemoglobin 16 g/dL, hematocrit 46.8%, mild acute renal failure with creatinine 1.5 mg/dL, elevated C-reactive protein 24.4 mg/dL, sedimentation rate 39 mm/hr, and elevated high-sensitivity troponin I of 72 (peak) declining to 62 ng/L. His respiratory viral panels were all negative. Bedside cardiac ultrasound revealed a small pericardial effusion without cardiac tamponade, moderately reduced ejection fraction (EF 40%; prior EF 45% in September 2022), and inferior vena cava (IVC) with normal respiratory variation (Fig. 2a-b, Supplementary Files 1–3). His chest x-ray showed atelectasis, and his chest computed tomography angiogram (CTA) showed a mild to moderate pericardial effusion with pericardial fat stranding but no pulmonary embolism or lung opacities (Fig. 2c-d).

We firstly considered acute coronary syndrome (ACS) with chest pain and ST segment elevations on ECG. Pericarditis was also highly likely with positional pleuritic chest pain, pericardial effusion on ultrasound, and diffuse ST segment elevations on ECG. However, the moderately reduced EF (40%) on ultrasound and elevated troponin I level were concerning for myocardial involvement, thus additional emergent workup was pursued. Differential diagnoses included acute congestive heart failure, aortic dissection, pneumonia, or a viral respiratory illness with his rhinorrhea, cough, and sore throat. Nevertheless, imaging was negative for bacterial pneumonia, and respiratory viral panels were negative. Chest CTA scan did not show pulmonary embolism or other lung pathology (Supplementary File 4).

Cardiology was consulted, who recommended treating the patient for myopericarditis based on clinical symptoms. They had low suspicion for ACS due to the reported respiratory symptoms with congestion and cough and lack of exertional chest pain, thus additional ACS workup including advanced cardiac imaging was not pursued. Per cardiology recommendations, the patient was treated for acute myopericarditis with 15 mg intravenous ketorolac, colchicine 0.6 mg orally twice daily due to his reduced glomerular filtration rate (GFR ~ 45) for 3 months, and high-dose ASA (aspirin) 650 mg three times daily for 3–4 weeks, with the duration to be determined based on symptom improvement. His rivaroxaban was held during aspirin treatment to decrease bleeding risk and because of his lower stroke risk (CHADS2-Vasc score of 2 for age). Cardiology also discontinued his dronedarone during colchicine treatment and initiated metoprolol succinate for atrial fibrillation/flutter rate control.

While being monitored overnight in the hospital’s clinical observation unit, the patient’s chest pain and dyspnea significantly improved within 24 h. Transthoracic echocardiogram the next day confirmed a small pericardial effusion, reduced ejection fraction of 40%, moderate mitral and tricuspid regurgitation, and a prolapsed mitral valve leaflet (unchanged from prior echocardiogram except for the new pericardial effusion). He was discharged home that day with exercise precautions including avoiding any strenuous physical activity, continuing high-dose ASA for 3–4 weeks, colchicine for 3 months, and cardiology follow up in 1–2 weeks [3]. Corticosteroids were not prescribed because of the patient’s heart failure and increased myocardial rather than pericardial involvement. Nevertheless, at his one-week post-discharge follow up appointment, he had developed an acute heart failure exacerbation due to persistent atrial fibrillation, despite achieving rate-control with metoprolol, so his cardiologist prescribed low-dose furosemide for six days. His physician planned for anticoagulation resumption, amiodarone initiation, and possible synchronized cardioversion in 3–4 weeks after his acute illness improved. His repeat transesophageal echocardiogram four weeks after hospital discharge showed an unchanged reduced ejection fraction of 40% with persistent known leaky mitral valve leaflet causing moderate mitral regurgitation (which was unrelated to his myopericarditis illness). He did not undergo a synchronized cardioversion for his atrial flutter/fibrillation because his heart rate was controlled with metoprolol and his myopericarditis symptoms were greatly improved. He was started on amiodarone and resumed his rivaroxaban for his atrial fibrillation/flutter, and his mitral valve was managed conservatively due to symptom improvement. The patient timeline is presented in Supplementary File 5.

## Conclusions

The strengths of this case report are the clinical considerations for diagnostic imaging and the treatment decision algorithms used for mild to severe cases of pericarditis versus myocarditis [6–7]. Both cardiac POCUS and CT imaging are used to help differentiate the patient’s myopericarditis from other acute emergent pathologies such as ACS, PE, aortic dissection, or pulmonary infection. Use of bedside POCUS can identify cardiac wall motion abnormalities in cases of ACS or more global changes such as reduced ejection fraction in acute heart failure, which guides management. Furthermore, POCUS can help avoid unnecessary heart catheterization, which is invasive, by identifying a pericardial effusion in some cases, supporting the diagnosis of myopericarditis [1–2]. The case report describes early tailored management with colchicine and high-dose ASA per ESC and AHA guidelines and coordination of treatment with cardiology for his atrial fibrillation/flutter.^4–5^ The paper is limited by the study design as a case report, only reporting the outcomes of a single patient rather than a larger cohort, which can vary based on the patient’s underlying medical history and additional illnesses. Its generalizability is limited by the single center, which may use differing treatment algorithms than other hospitals. Potential study center bias is mitigated by citing national and international cardiology guidelines (ESC, AHA) to guide diagnosis and treatment decisions [4–5].

Although myopericarditis is uncommon, it is important to differentiate it from pericarditis or ACS as it can cause permanent heart damage and severe illness. Studies involving military personnel and healthy young adults have shown myopericarditis after smallpox vaccination and COVID-19 vaccination but not with influenza vaccination [7]. In another study, 14.6% of admitted patients with pericarditis had myocardial involvement, with 60% having a recent viral infection [3, 7]. 

Physicians should evaluate for ACS in all patients presenting with chest pain, as ACS closely mimics myopericarditis and is a critical time-sensitive illness [3]. The distinguishing diagnostic criteria for myocarditis include elevated cardiac biomarkers and left ventricular dysfunction on echocardiogram. Cardiac POCUS can reveal complications such as a large pericardial effusion, left ventricular dysfunction, or cardiac tamponade and guide therapeutic paracentesis or diuresis if needed [1–2]. Our patient had left ventricular dysfunction on echocardiogram with a moderately reduced EF 40%, indicating higher illness severity with myocardial involvement and potential worsening from his atrial flutter. POCUS can also help evaluate for other chest pain pathologies such as enlarged right ventricle (RV), septal bowing, and McConnell’s sign (RV free wall akinesis with apical sparing) in cases of acute pulmonary embolism or right heart strain; aortic dissection flap; aortic or left ventricular aneurysm; congestive heart failure; or cardiac tamponade (right atrial systolic collapse and diastolic RV collapse) [7–8].

Cardiac MRI provides both morphological and hemodynamic information and is helpful in unclear or recurrent cases of pericarditis [1, 3]. In patients with myocarditis, cardiac MRI will demonstrate subepicardial or mid-myocardial inflammation with late gadolinium enhancement (94% sensitivity) and myocardial edema in *different* vascular territories (whereas ACS shows myocardial enhancement in a *single* arterial territory) [1, 3]. The gold diagnostic standard for myocarditis is endomyocardial biopsy, but this is invasive and not performed in all cases.

Correct myopericarditis diagnosis is important as the inflammation can progress to permanent myocardial damage causing cardiomyopathy, arrhythmias, and heart failure without appropriate early therapeutics. Per the 2025 ESC and AHA guidelines for the management of myocarditis and pericarditis, cardiology consultation and early treatment with nonsteroidal anti-inflammatories, colchicine, and high-dose ASA (650 mg three times daily) is recommended for myopericarditis [4–5]. Exercise is restricted to walking, light weight training, and no active sports for three months. Treatment is complete after symptom resolution and normalization of inflammatory markers [8]. 

Clinical outcomes are typically excellent, with most patients fully recovering within one month with normal cardiac function. One study showed that up to 14% of patients may still report mild chest discomfort [2]. In another review of 389 patients, 3.5% had residual left ventricular dysfunction without heart failure at 31 months. If inflammation persists over time, then scar tissue increases the risk for arrhythmias and cardiac dysfunction. The AHA scientific statement reports that up to 30% of biopsy-proven myocarditis can transition into chronic inflammatory cardiomyopathy [5, 9]. While patients with nonfulminant/uncomplicated myocarditis have almost no cardiac mortality, patients presenting with fulminant myocarditis (cardiogenic shock) or arrhythmias can have 10% mortality at 30 days and a 15% heart transplant rate at 5 years [10]. Thus, early treatment of inflammation, cardiac arrhythmias, and acute heart failure symptoms can help prevent permanent heart damage or complications. Finally, studies report that over 90% of recurrent cases presented as pericarditis, with cardiac tamponade and constrictive pericarditis comprising < 1% of cases [7–8].

In conclusion, patients with myopericarditis are risk stratified by illness severity. Patients with more myocardial involvement can develop complications such as heart failure or cardiac arrhythmia benefiting from emergent treatment. Diagnostic imaging includes cardiac POCUS and CT or MRI imaging to help differentiate between myopericarditis, ACS, and PE and identify complications such as cardiac tamponade. Myopericarditis treatment includes nonsteroidal anti-inflammatories, colchicine, and high-dose ASA, and patients can exhibit excellent recovery with early treatment.

### Patient’s perspective

The road to [name redacted] Emergency Department started by first going to Urgent Care with severe chest pain, headache, sore throat, and trouble breathing when lying down. I thought I had COVID-19, but both rapid and long tests were negative. Urgent Care gave me an antibiotic and cough syrup and told me I was at risk for pneumonia with some fluid in my lungs. I followed up with my primary care doctor the next day. He performed an ECG and blood tests. He told me after seeing the results of the tests to go directly to the Emergency Room because he thought I might be having a heart attack. When I got to the Emergency Department, the triage team took me [back to a room] right away. I was given IV medications that improved my breathing and overall symptoms of chest discomfort. I was relieved to hear that I was not having a heart attack, and I was quickly diagnosed with myopericarditis. I took a battery of tests (chest x-ray, CT scan, echocardiogram) over the next 24 h [while being continuously monitored in the Clinical Observation Unit]. I felt that my symptoms were 85% improved by the next morning after the medication treatment was started. I was discharged the next day with follow-up medications and medical appointments. Despite the medications I was taking at home, I experienced severe left shoulder and chest pain, coughing, headache, trouble breathing, and malaise for the next two weeks after leaving the Emergency Room.

## Supplementary Information

Below is the link to the electronic supplementary material.

Supplementary Material 1.

## Data Availability

No datasets were generated or analysed during the current study.

## Abbreviations

ECG: Electrocardiogram (ECG)
ACS: Acute coronary syndrome
EF: Ejection fraction
ASA: Aspirin
ED: Emergency department
COVID-19: Coronavirus disease
CT: Computed tomography
MRI: Magnetic resonance imaging
CARE: Guidelines (for Case Reports)
JVD: Jugular venous distension
IVC: Inferior vena cava
CTA: Chest computed tomography angiogram
POCUS: Point-of-care ultrasound
RV: Right ventricle
