# Supplementary material for: Myopericarditis in an emergency department patient presenting with chest pain and ECG changes: a case report
Source: Egypt Heart J. 2026 Apr 7;78:22. doi: 10.1186/s43044-026-00734-7 (PMC13057090; doi:10.1186/s43044-026-00734-7)
Supplement: Supplementary file 1 — Supplementary Material 1. [file 43044_2026_734_MOESM1_ESM.zip › Supp File 5 Patient care timeline.docx]

Supplementary File 6: Patient care timeline

Key: Emergency Department (ED), HsTNT (high sensitivity troponin I), left ventricle (LV), ASA (aspirin), acute coronary syndrome (ACS), pulmonary embolism (PE)
