# Supplementary figures and images for: Myopericarditis in an emergency department patient presenting with chest pain and ECG changes: a case report
Source: Egypt Heart J. 2026 Apr 7;78:22. doi: 10.1186/s43044-026-00734-7 (PMC13057090; doi:10.1186/s43044-026-00734-7)

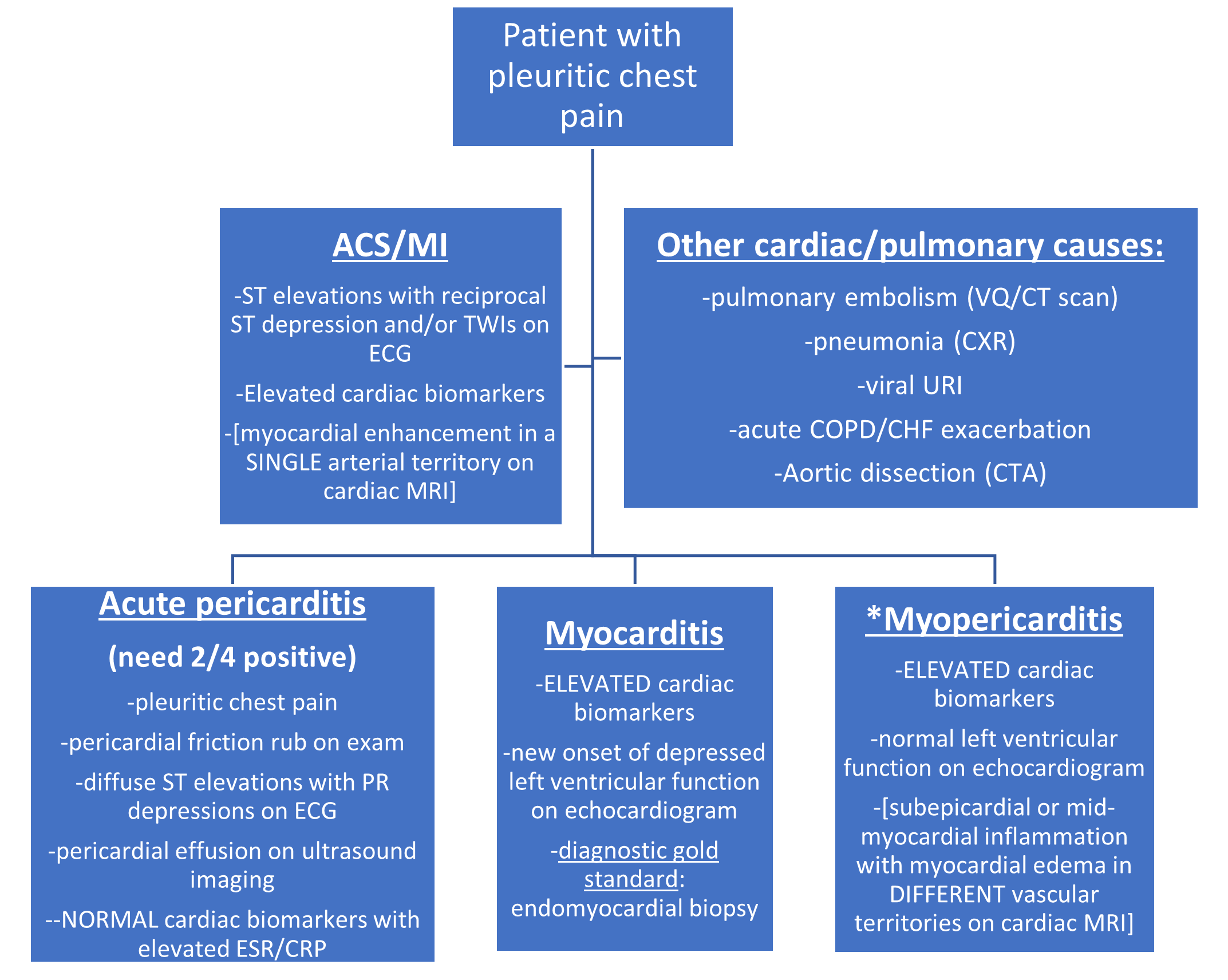

Supplement: Supplementary file 1 — Supplementary Material 1. [file 43044_2026_734_MOESM1_ESM.zip › 1-Supp File 4 Myopericarditis diff dx300.tif]
